# Differential Contributions of MYCs to Insect Defense Reveals Flavonoids Alleviating Growth Inhibition Caused by Wounding in *Arabidopsis*

**DOI:** 10.3389/fpls.2021.700555

**Published:** 2021-07-13

**Authors:** Dan-Dan Wang, Pai Li, Qiu-Yi Chen, Xue-Ying Chen, Zi-Wei Yan, Mu-Yang Wang, Ying-Bo Mao

**Affiliations:** ^1^CAS Key Laboratory of Insect Developmental and Evolutionary Biology, CAS Center for Excellence in Molecular Plant Sciences, Shanghai Institute of Plant Physiology and Ecology, Chinese Academy of Sciences, Shanghai, China; ^2^School of Life Sciences and Technology, ShanghaiTech University, Shanghai, China; ^3^University of Chinese Academy of Sciences, Beijing, China

**Keywords:** MYC transcription factors, transcriptome, plant resistance to insects, WGCNA, flavonoids

## Abstract

In *Arabidopsis*, basic helix–loop–helix transcription factors (TFs) MYC2, MYC3, and MYC4 are involved in many biological processes, such as defense against insects. We found that despite functional redundancy, MYC-related mutants displayed different resistance to cotton bollworm (*Helicoverpa armigera*). To screen out the most likely genes involved in defense against insects, we analyzed the correlation of gene expression with cotton bollworm resistance in wild-type (WT) and MYC-related mutants. In total, the expression of 94 genes in untreated plants and 545 genes in wounded plants were strongly correlated with insect resistance, and these genes were defined as MGAIs (MYC-related genes against insects). MYC3 had the greatest impact on the total expression of MGAIs. Gene ontology (GO) analysis revealed that besides the biosynthesis pathway of glucosinolates (GLSs), MGAIs, which are well-known defense compounds, were also enriched in flavonoid biosynthesis. Moreover, MYC3 dominantly affected the gene expression of flavonoid biosynthesis. Weighted gene co-expression network analysis (WGCNA) revealed that *AAE18*, which is involved in activating auxin precursor 2,4-dichlorophenoxybutyric acid (2,4-DB) and two other auxin response genes, was highly co-expressed with flavonoid biosynthesis genes. With wounding treatment, the WT plants exhibited better growth performance than chalcone synthase (*CHS*), which was defective in flavonoid biosynthesis. The data demonstrated dominant contributions of MYC3 to cotton bollworm resistance and imply that flavonoids might alleviate the growth inhibition caused by wounding in *Arabidopsis*.

## Introduction

Because of their sessile lifestyle, plants have developed numerous strategies to cope with biotic and abiotic stresses from the environment, such as insect herbivores and pathogenic infections (Howe and Jander, 2008). Jasmonates (JAs) are the main regulators involved in defense response and plant development (Erb and Glauser, 2010; Okada et al., 2015; Chen et al., 2019; Wasternack and Strnad, 2019; Zander et al., 2020). The biosynthesis and signal transduction of JAs have been extensively studied in the past decades (Browse, 2009; Erb et al., 2012; Wasternack and Hause, 2013; Howe et al., 2018).

MYC transcription factors (TFs) that have a conserved basic helix-loop-helix (bHLH) motif, are the main regulators of the JA signaling pathway. Although MYC2, MYC3, and MYC4 have redundant functions, evidence has shown their differential contributions in *Arabidopsis* (Carretero-Paulet et al., 2010; Goossens et al., 2017; Wang et al., 2019). MYC2 is considered as the master regulator of most aspects of JA signaling transduction (Kazan and Manners, 2013; Yang et al., 2019), whereas MYC3 and MYC4 have specificity similar to that of MYC2 to bind the cis-element G-box, which assists in the activation of a gene expression (Fernandez-Calvo et al., 2011).

MYC TFs regulate the expression of a series of secondary metabolic pathways, such as glucosinolates (GLSs), flavonoids, and alkaloids (De Geyter et al., 2012; Zhou and Memelink, 2016; Chen et al., 2019; Wasternack and Strnad, 2019). GLS are important defense compounds in *Brassicaceae*, containing sulfur- and nitrogen-thioglucosides (Bones and Rossiter, 2006; Halkier and Gershenzon, 2006; Manzaneda et al., 2010). MYC2, MYC3, and MYC4 regulate GLS biosynthesis by directly interacting with MYB TFs (Schweizer et al., 2013b). Plant polyphenolic secondary metabolites, flavonoids, are synthesized from the phenylpropanoid pathway and are considered as antioxidants that relieve oxidative stress in plants (Agati et al., 2012; Petrussa et al., 2013; Wen et al., 2020). Chalcone synthase (CHS) catalyzes the formation of a molecule of p-coumaroyl-CoA with three molecules of malonyl-CoA to form naringenin chalcone (4,2,4,6-tetrahydroxychalcone), which is the first step in the core flavonoid synthesis pathway (Jiang et al., 2015). Accumulation of flavonoids in plants has been observed in response to various stresses (Chalker-Scott, 1999; Stracke et al., 2010; Clayton et al., 2018; Liang et al., 2020). Flavonoids have diverse functions in regulating plant physiology, for example, as negative regulators of auxin transport in *Arabidopsis* (Brown et al., 2001; Tan et al., 2019; Sharma et al., 2020; Dong and Lin, 2021). In some cases, extremely high concentrations of flavonoids are thought to be toxic to insects (Simmonds and Stevenson, 2001; Anshul et al., 2013; Onkokesung et al., 2014).

MYC TFs are well-known regulators of plant defense against insects. The triple mutant *myc2 myc3 myc4* (*mycT*) is hypersensitive to the generalist herbivore *Spodoptera littoralis* (Fernandez-Calvo et al., 2011; Schweizer et al., 2013a). Of the redundant target genes regulated by MYCs, the direct defense genes and the differential contributions of MYC2, MYC3, and MYC4 in plant defense against herbivorous insects have not been systemically studied. Here, we found that *myc*-related mutants have different resistance to generalist herbivores (*Helicoverpa armigera*). Using weighted gene co-expression network analysis (WGCNA), we screened out 626 genes, of which the expressions were highly correlated with insect resistance; and further, they were named as MGAIs (MYC-related genes against insects). MYC3 contributes more to MGAIs expression than MYC2 and MYC4. MGAIs were significantly enriched in GLS and flavonoid biosynthesis. We further found that flavonoids alleviate the growth inhibition caused by wounding treatment in *Arabidopsis*. The data elaborated the differential contributions of MYCs to insect resistance and provided a possible role for flavonoids in plant defense.

## Materials and Methods

### Plant and Insect Cultures

*Arabidopsis thaliana* (Ecotype Col-0) was grown under long-day photoperiod (16-h light/8-h dark) conditions at 22°C. The *myc*-related mutants, namely, the single mutants *myc2-2* (SALK_083483), *myc3* (GK445B11), and *myc4* (GK491E10); double mutants (*myc2 myc3*, *myc2 myc4*, and *myc3 myc4*); triple mutants (*myc2 myc3 myc4* and *mycT*), and *chs* (SALK_020583) have been described previously (Fernandez-Calvo et al., 2011; Song et al., 2014; Zhang et al., 2021).

The larvae of cotton bollworm (*H. armigera*) and diamondback moth (*Plutella xylostella*) were obtained from the Institute of Zoology, Chinese Academy of Sciences, and reared on an artificial diet at25°C with 70% relative humidity and a 14-h light/10-h dark photoperiod. For insect feeding tests, second instar *H. armigera* larvae and *P. xylostella* larvae 2 days post hatching were reared on *Arabidopsis* leaves (replaced with fresh leaves every day) for 4 days and weighed.

### Intermittent Wounding Treatment

After the plants were grown for 12 days, the leaves were wounded for the first time using microtips. The newly grown leaves were subsequently wounded in the same way every 3 days. After 3–4 rounds of the treatment, the aerial portions were harvested and used for biomass detection.

### RNA Extraction, Reverse Transcription, and Quantitative Real-Time PCR

Total RNA was extracted from *Arabidopsis* using a TRIzol reagent (Invitrogen, Waltham, MA, United States). One microliter of DNase I (40 U/μl, Promega, Madison, WI, United States) was added to total RNA to remove genomic DNA, and1 μg of total RNA was used to prepare the first strand cDNA (Transgene, Beijing, China). Real-time polymerase chain reaction (qRT-PCR) was performed with a Bio-Rad CFX Connect qRT-PCR system (Bio-Rad, United States) using SYBR Green PCR Mix (Takara, Japan), according to the instructions of the manufacturer for the standard step amplification program. *Arabidopsis S18* (*At4g09800*) was used as an internal standard. Biological triplicates were performed with technical duplicates. All nucleotide primers used in this study are listed in Supplementary Table 1.

### *Arabidopsis* Sample Preparation and RNA Sequencing

For transcriptome sequencing, the second pair of true leaves of the 18-day wild-type (WT) (Col-0) and *myc*-related mutants (*myc2-2*, *myc3*, *myc4*, *myc2/3*, *myc2/4*, *myc3/4*, and *mycT*) were wounded and harvested 4 h post wounding. The corresponding untreated plants were used as controls (CK). Each sample contained three biological replicates. Total RNA was extracted and approximately 1 μg of total RNA was used to enrich poly(A) mRNA using oligo-dT magnetic beads (Invitrogen, Waltham, MA, United States), followed by fragmentation into 100–400 nt sizes; and the fragments were subsequently used to synthesize cDNA with random hexamer primers (Invitrogen, Waltham, MA, United States). RNA sequencing was performed on an Illumina HiSeqXten platform (Illumina, San Diego, CA, United States) at Majorbio (Shanghai, China).

### Glucosinolates Analysis

Glucosinolates were detected as previously described (Glauser et al., 2012). The 18-day-old *Arabidopsis* leaves were immediately frozen in liquid nitrogen after harvest and lyophilized to dryness for 24 h. The dry samples (10–20 mg) were extracted with 2 ml of 70% methanol (v/v), with 0.1 μmol internal standard (sinigrin) and then incubated at 80°C for 15 min. The samples were centrifuged after cooling. The supernatants were concentrated in a vacuum to a final volume of 250 μl and filtered using a syringe filter before being used for liquid chromatograph-mass spectrometer (LC-MS) analysis. The prepared samples (5 μl) were analyzed using an Agilent 1290 Infinity LC pump (Agilent, Santa Clara, CA, United States) and by 6125 single quadrupole mass spectrometry. The analysis conditions were as follows: HPLC: column: ACQUITY UPLC CSH C18 (pore size 1.7 μm, length 2.1 × 100 mm); solvent system: acetonitrile (0.1% formic acid), water (0.1% formic acid); gradient program, 0 min: 2:98 v/v, 6 min: 45:55 v/v, 6.5 min: 100:0 v/v, 8.5 min: 100:0 v/v; flow rate, 0.4 ml/min; temperature, 25°C. Mass spectrometry detection: capillary voltage, 3,000 V; air temperature, 350°C; dry gas flow rate, 12 L/min. LC-MS based metabolomics was performed using the negative ion mode (Mz 100–1,000).

### Statistical Analysis

#### Differential Gene Expression Analysis

Differentially expressed genes (DEGs) were determined by comparing expression levels in WT and *myc*-related mutants using the R package DESeq2 (Love et al., 2014). Only genes with relatively high expression levels (mean count > 1) were subjected to further analysis. Genes with a log2 fold change greater than 1 and adjusted *P*-value less than 0.05 in Bonferroni correction were defined as DEGs.

#### Correlation Calculation and MGAI Identification

We used the mean weights of cotton bollworms fed on *myc*-related mutants to reflect the resistance of the corresponding plants. We calculated the Pearson correlation between cotton bollworm resistance (opposite numbers to average of weight of insects) and gene expression in different plants and tested the results by Student’s *t*-test. We chose the genes with a correlation greater than 0.6 and a *P* value less than 0.05 as MGAIs.

#### Co-expressed Analysis

Weighted gene co-expression network analysis was performed to generate an unsigned co-expression network on all sample data based on topology overlap measurement (TOM) using the R package WGCNA (Langfelder and Horvath, 2008). Genes with an average TPM (transcripts per million) greater than one among all samples were used to generate the network. The soft threshold β was chosen to be 18. All the genes were clustered into 55 different modules (and a gray module containing genes that could not be classified to any module) by hierarchical clustering using a distance matrix (1-TOM).

#### Enrichment Analysis

All enrichment analyses were based on Fisher’s exact test with Bonferroni correction. Gene ontology (GO) enrichment analysis was performed using GO annotations downloaded from TAIR^1^. GO enrichment, Kyoto Encyclopedia of Genes and Genomes (KEGG) enrichment, and module enrichment analyses were performed using the R package cluster Profiler (Yu et al., 2012) and KEGGREST (Tenenbaum and Maintainer, 2020).

## Results

### The *myc*-Related Mutants Display Differential Resistance to Cotton Bollworm

**FIGURE 1:**
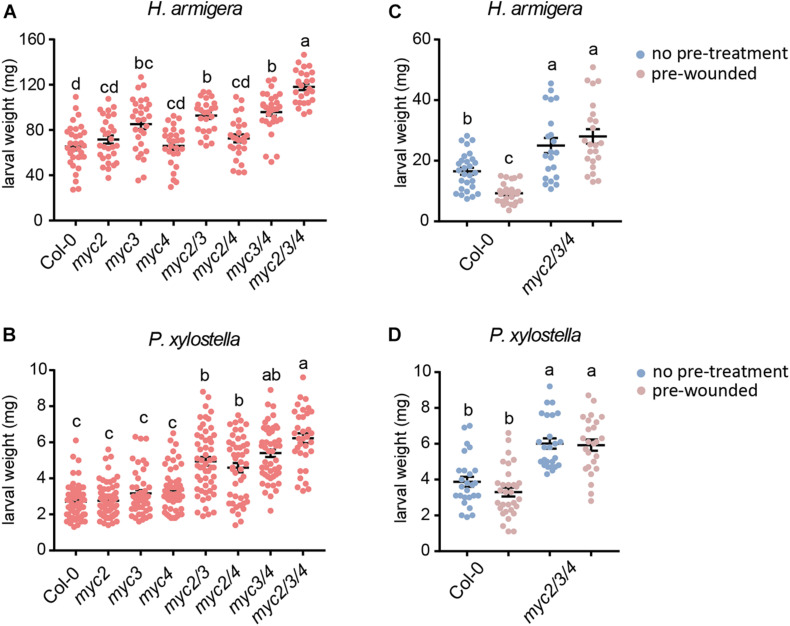
Involvement of MYC2, MYC3, and MYC4 in plant resistance to insect. *Helicoverpa armigera* **(A,C)** and *P. xylostella* **(B,D)** larvae were fed with the indicated plants for 4 days. In panels **(C,D)**, the plants were wounded 1 day before being used for feeding. Data are means ± SEM (*n* = 25–35) analyzed by one-way ANOVA and Tukey’s honest significant difference (HSD) test. Different letters indicate significant differences, *P*-value < 0.05.

To clarify the differential contributions of MYC2, MYC3, and MYC4 in plant defense against herbivorous insects, we tested the resistance of the *myc*-related mutants to that of the generalist insect, *H. armigera*, and the specialist insect, *P. xylostella*. For *H. armigera* larvae, the single mutant *myc3* and the *myc3* related double mutants (*myc2 myc3*, *myc3*, and *myc4*) displayed attenuated resistance, and the attenuation was more serious in the triple mutant (*myc2 myc3 myc4*, and *mycT*); however, the resistance of the other single mutants (*myc2* and *myc4*) and double mutant (*myc2* and *myc4*) were similar to that of the WT (Figure 1A). None of the single mutants (*myc2*, *myc3*, and *myc4*) showed any obvious change in resistance to *P. xylostella* larvae. All the double mutants displayed reduced resistance; however, no obvious differences among the double mutants were observed, and *mycT* displayed the highest sensitivity to *P. xylostella* larvae (Figure 1B). These results indicated that MYC3 played a dominant role in resistance to cotton bollworm, while the three MYCs had highly redundant functions in resistance to *P. xylostella*.

Many plant genes are induced by wounding damage caused by chewing insects and are thought to be important in insect defense. We used wounding pre-treated plants (24 h post wounding) for insect feeding to test whether wounding pre-treatment could improve plant resistance to insects. When plants were pre-treated with wounding, the resistance of WT but not *mycT* to *H. armigera* was enhanced but not *mycT* (Figure 1C). However, the resistance to *P. xylostella* was barely affected by pretreatment with wounding in either WT or *mycT* (Figure 1D). These results indicate that the enhanced resistance to *H. armigera* caused by wounding is regulated by MYCs.

### MYC2, MYC3, and MYC4 Regulate Different Biological Processes

**FIGURE 2:**
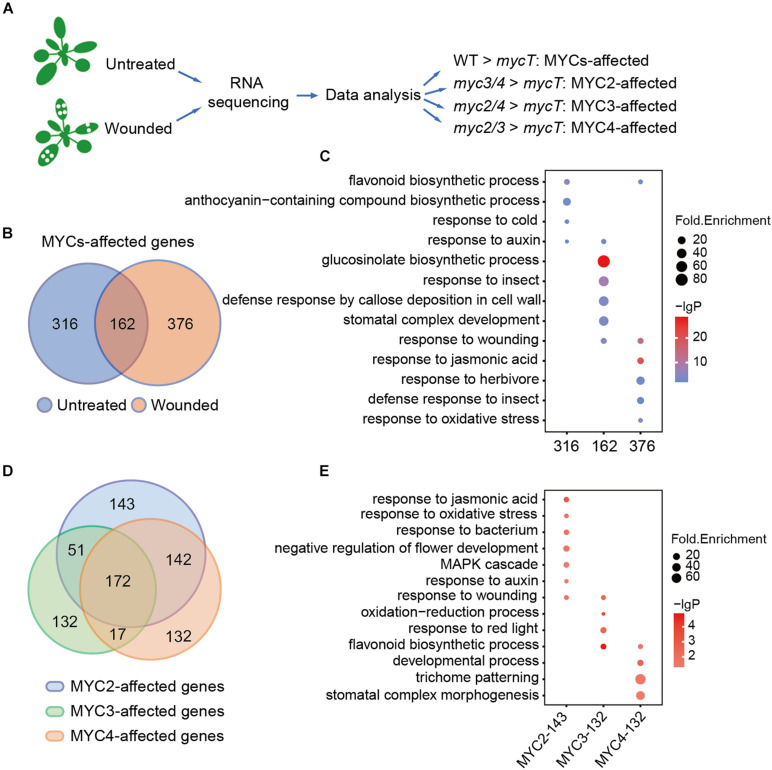
MYC2, MYC3, and MYC4 have different contributions in biological processes. **(A)** Workflow of sample collection, sequencing, and analysis. The untreated plants and the plants 4 h post wounding were collected and used for RNA sequencing and data analysis. **(B)** Venn diagram shows MYC-affected genes in the plants with (orange and wounded) or without (blue and untreated) wounding treatment. **(C)** GO enrichment analysis of 316, 162, and 376 MYC-affected genes as described in panel **(B)**. **(D)** Venn diagram of MYC2-, MYC3-, and MYC4-affected genes in either untreated or wounded plants, which were described in Supplementary Figure 2A. **(E)** GO analysis of genes only affected by MYC2, MYC3, and MYC4, respectively. **(C,E)** The size of the dot represents fold enrichment, and color stands for –lg(*P*-value).

To gain a deeper insight into the plant defense regulated by MYCs, RNA-seq data of the WT and *myc*-related mutants both with and without wounding were analyzed. Owing to the redundant function of MYCs, we compared double the mutants (*myc2*, *myc3*, *myc2*, *myc4*, *myc3*, and *myc4*) with *mycT* to analyze the effects of single MYC on gene regulation. Genes with significantly higher expressions in *myc3* and *myc4* than in *mycT* were defined as the MYC2-affected genes, and so on, to the MYC3- and MYC4-affected genes. Genes that were significantly higher in the WT than in *mycT* were defined as MYC-affected genes (Figure 2A).

Without wounding, the expression of 478 genes was significantly higher in WT than in *mycT*, and with the wounding treatment, 538 genes were found to have higher expression in WT than in *mycT*. Venn diagram analysis revealed 162 overlapping genes (Figure 2B and Supplementary Table 2). The 316 MYC-affected genes from the untreated samples were enriched in flavonoid and anthocyanin biosynthesis processes. The overlapping genes were enriched in “GLS biosynthetic process” and those only from the wounded sample analysis were mainly enriched in “response to jasmonic acid” (Figure 2C and Supplementary Table 3). These results revealed a general profile of MYC-regulated genes. We selected six MYC-affected genes and analyzed their expression in different mutants by RT-qPCR, and the results are consistent to those of RNA-seq assay (Supplementary Figure 1).

Comparisons of gene expression in double mutants and *mycT* both with and without wounding revealed 508 MYC2-affected, 372 MYC3-affected, and 463 MYC4-affected genes (Supplementary Figure 2A and Supplementary Table 4). A total of 172 genes, whose expression was affected by all the three MYCs (Figure 2D), were significantly enriched in “GLS biological process” (Supplementary Figure 2B and Supplementary Table 5), suggesting functional redundancy of MYCs in this process. Genes only affected by MYC2 (132) were enriched in “response to jasmonic acid” and “response to auxin,” whereas genes only affected by MYC3 (143) were enriched in “flavonoids biosynthetic process” and “response to red light”; and genes only affected by MYC4 (132) were enriched in “trichome patterning” (Figure 2E and Supplementary Table 5). These results suggest that despite functional redundancy, MYCs differentially regulate gene expression in multiple biological processes.

### MYC3 Has the Most Significant Impact on the Expression of Defense Genes Against Cotton Bollworm

**FIGURE 3:**
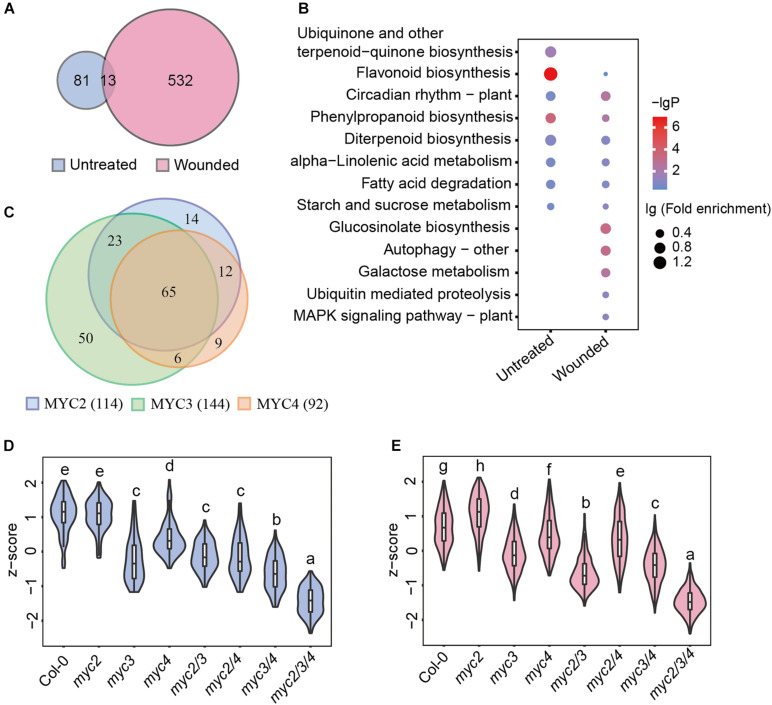
MYC3 has the most significant impacts on the MGAI expressions. **(A)** Venn diagram of MGAIs in untreated (blue) and wounded (red) plants. **(B)** GO enrichment analysis of MGAIs as described in panel **(A)**. The dot represents lg (fold enrichment), and color stands for –lg(*P*-value). **(C)** Venn diagram of MYC2-, MYC3-, and MYC4-affected MGAIs. Blue, green, and orange, respectively, stand for the total MGAIs affected by MYC2, MYC3, and MYC4 in untreated and wounded plants as described in Supplementary Figure 5. **(D,E)** Expressions of the MGAIs obtained from the untreated **(D)** and the wounded **(E)** plants, as described in panel **(A)**. The TPM of each gene was normalized into *z*-score. The detailed MGAI information is listed in Supplementary Table 11. Data are analyzed by one-way ANOVA and Tukey’s HSD test. Different letters indicate significant differences, *P*-value < 0.05.

In addition to defense genes, MYCs also regulate multiple genes involved in growth, development, etc. To screen for MYC-regulated genes in defense against cotton bollworm, we analyzed the correlation between gene expression and cotton bollworm resistance in WT and *myc*-related mutants. The expression of 94 genes in untreated plants and 545 genes in wounded plants were found to be strongly correlated with insect resistance (Pearson correlation coefficient, *r* > 0.6) (Supplementary Table 6), and these genes were termed as MGAIs (Supplementary Figure 3). Interestingly, the Venn diagram showed that only 13 MGAIs in untreated and wounded plants overlapped (Figure 3A and Supplementary Table 7). MGAIs from the untreated samples were significantly enriched in the “flavonoid biosynthesis process,” whereas those from the wounding treated samples were enriched in “GLS biosynthetic process” (Figure 3B and Supplementary Table 8), consistent with a previous study showing that GLSs are well-known anti-insect compounds (Wittstock and Gershenzon, 2002; Mewis et al., 2006; Schlaeppi et al., 2008). GLS synthetic gene expression was largely downregulated in *mycT* but was not significantly reduced in single or double mutants (Supplementary Figure 4A and Supplementary Table 9), which further confirmed the redundant function of the three MYCs in the regulation of GLS biosynthesis. RT-qPCR analysis of the selected GLS synthesis genes was consistent with the RNA-seq results (Supplementary Figure 4B). Furthermore, the GLS content in the plants was highly correlated with their resistance to the cotton bollworm (Supplementary Figures 4C,D).

Of the 626 total MGAIs, the expressions of 273 genes were remarkably affected by MYCs, for their expression in either untreated or wounding-treated *mycT* was significantly downregulated (Supplementary Figure 5 and Supplementary Table 10), and the number of MYC3-affected MGAIs (144) were more than MYC2(114)- and MYC4(92)-affected genes (Figure 3C, Supplementary Figure 5, and Supplementary Table 10). For the 94 MGAIs in the untreated plants, the total expression levels were significantly reduced in *mycT*; and notably, of the three single mutants, the lowest expressions were observed in *myc3* (Figure 3D and Supplementary Table 11). Similar trends in gene expression were observed for the 545 MGAIs in wounded plants (Figure 3E and Supplementary Table 11). These results suggest the dominant function of MYC3 on the expression of MGAIs in either untreated or wounded plants.

### Classification of Gene Modules Highly Correlated With Plant Resistance to Cotton Bollworm

**FIGURE 4:**
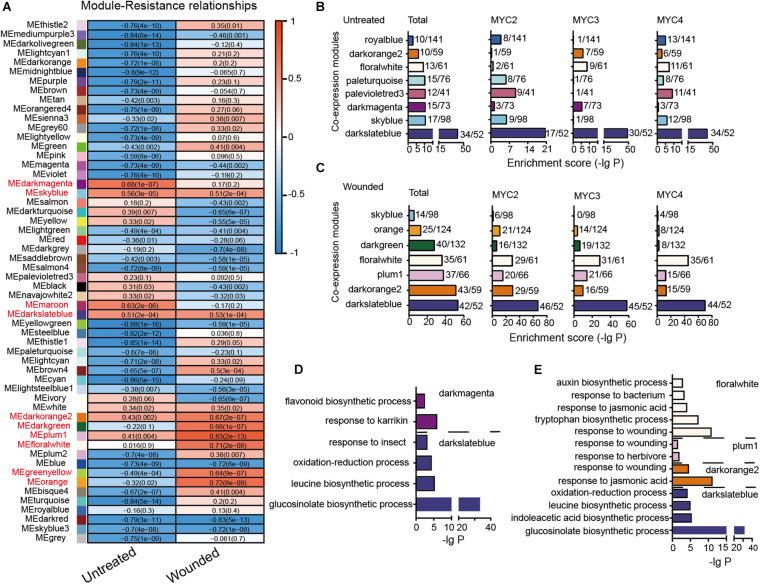
Module classification based on gene co-expression. **(A)** Correlation of the module eigengenes with cotton bollworm resistance. Red to blue stands for positive to negative correlations between the module eigengenes and the plant resistance to cotton bollworm. The numbers represent correlation coefficients; and the numbers in brackets stand for *P*-value. **(B,C)** MYC2-, MYC3-, and MYC4-affected genes of the untreated **(B)** and wounded **(C)** plants are enriched in the indicated modules. The a/b values above the bars indicate gene numbers affected by all the three MYCs (Total) or the indicated MYC (a) and gene numbers of the corresponding module (b). **(D,E)** GO analysis of MYC-affected genes from untreated **(D)** and wounded **(E)** plants that were distributed in the indicated modules.

Genes with highly correlated expression are helpful in determining their association with biological processes. A total of 16,660 genes with an average TPM value greater than one across all 48 tested groups were subjected to WGCNA. In total, 10,991 genes were divided into 55 modules (Supplementary Figure 6). The correlation between the module eigengenes and cotton bollworm resistance is presented in Figure 4A. The eigengenes of two modules (dark magenta and maroon) in untreated plants and of six modules (plum1, orange, floral white, dark green, dark orange 2, and green yellow) in the wounded plants were highly correlated with plant resistance (*r* > 0.6) (Figure 4A). The eigengenes of only two modules (sky blue and dark slate blue) in the untreated and wounded plants showed a similar positive correlation with resistance.

In the untreated plants, genes whose expression was affected by the three MYCs were mainly distributed across eight modules, such as dark magenta, of which eigengenes are highly correlated with resistance to cotton bollworm (Figures 4A,B). The distributions of MYC2-, MYC3-, and MYC4-affected genes across the eight modules were quite different, reflecting their distinct role in regulating gene expression in untreated plants. For example, the genes classified as dark magenta were primarily affected by MYC3 (Figure 4B and Supplementary Table 12) and were enriched in the flavonoid biosynthetic process (Figure 4D and Supplementary Table 13).

In the wounded plants, seven modules, such as dark slate blue, were significantly affected by MYCs. Interestingly, the distributions of MYC2-, MYC3-, and MYC4-affected genes across these seven modules were similar, indicating that the functions of MYC2, MYC3, and MYC4 on gene expression in wounding response are highly redundant (Figure 4C and Supplementary Table 12). The MYC-affected genes of dark orange 2, plum1, and floral white modules were all enriched in “the response to jasmonic acid” (Figure 4E and Supplementary Table 13), reflecting the dominant role of JA in the wounding response regulated by MYCs.

Furthermore, the total MYC-affected genes of dark slate blue in both untreated and wounded plants were enriched in the GLS biosynthetic process. The expression of dark slate blue in untreated and wounded plants was positively correlated with cotton bollworm resistance (Figure 4A), suggesting the important role of GLS in resistance against cotton bollworm in both untreated and wounded plants (Figures 4D,E).

### Flavonoid Synthesis and Growth-Related Genes Are Highly Co-expressed and Mainly Regulated by MYC3

**FIGURE 5:**
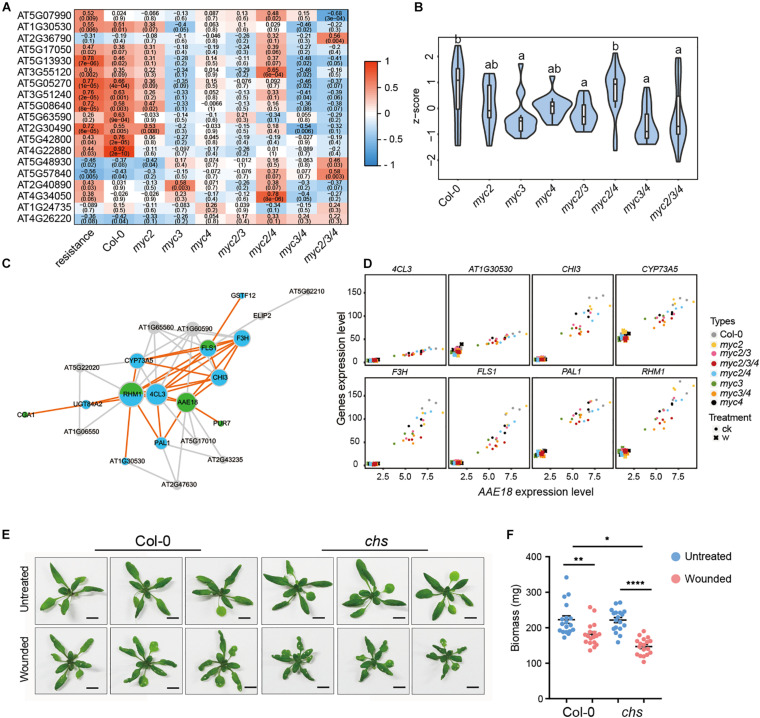
Flavonoids alleviate the growth inhibition caused by intermittent wounding. **(A)** Heatmap showing the effect of MYCs on the expressions of flavonoid synthesis genes positively associated with plant resistance. Red to blue represents the positive to negative correlation between gene expression and resistance. The number indicates correlation coefficient, and the number in brackets indicates *P*-value. **(B)** The violin plot of flavonoid gene expression in *myc*-related mutants in untreated plants. *Z*-score represent the normalized TPM of each gene. The detailed flavonoid gene information is presented in Supplementary Table 14. Data are analyzed by one-way ANOVA and Tukey’s HSD test. Different letters indicate significant differences, *P*-value < 0.05. **(C)** Co-expression network of flavonoid synthesis and growth-related genes. Blue and green stand for flavonoids synthesis genes and growth-related genes, respectively. **(D)** Scatter plot displays the co-expression of *AAE18* with the eight flavonoid synthesis genes. *X*-axis stands for the *AAE18* expressions (TPM), and *Y*-axis stands for the expressions (TPM) of the indicated genes in flavonoids synthesis. Dots represent the gene expressions in untreated samples, and crosses represent the gene expressions in wounding treated samples. **(E,F)** Images **(E)** and Biomass **(F)** of WT and *CHS* with intermittent wounding treatments. When plant has grown for 12 days, the first wounding treatment is performed, and the wounding treatment is performed every 3 days for a total of three times. **(E)** Scale bar, 1 cm. **(F)** Data are means ± SEM (*n* = 18) and analyzed by two-way ANOVA and Tukey’s HSD test. *P < 0.05, **P < 0.01, ****P < 0.0001.

In *Arabidopsis*, 19 genes were involved in flavonoid synthesis according to the KEGG annotation, and the expression of most genes was positively associated with resistance to cotton bollworm in untreated plants (Figure 5A). The expressions of most of these genes were found to be suppressed in all the MYC3-related mutants (*mycT*, *myc3 myc4*, and *myc3*) but not in *myc2*, *myc4*, and *myc2 myc4* (Figure 5A). Moreover, the total expression of flavonoid synthesis genes was significantly reduced in all the MYC3-related mutants. These results indicate that flavonoid synthesis genes were mainly regulated by MYC3 (Figure 5B and Supplementary Table 14). In addition to flavonoid synthesis genes, we noticed that some growth-related genes (*AAE18*, *PUR7*, and *CGA1*) were also classified in the dark magenta module (Supplementary Figure 7), of which *AAE18* (*Acyl-activating enzyme 18*) is involved in the metabolism of auxin precursors to active auxins (Wiszniewski et al., 2009). *CGA1* (*cytokinin-responsive GATA factor 1*) and *PUR7* (*purin 7*) are auxin response genes that are involved in plant growth (Senecoff et al., 1996; Zubo et al., 2018). These three genes were highly co-expressed with flavonoid synthesis genes (Figure 5C). We selected eight flavonoid synthesis genes to individually analyze their co-expression with *AAE18* using a scatter plot. The results, along with Pearson correlations, showed that the *AAE18* expression was highly correlated with all the selected genes (Figure 5D). This implies that flavonoids might act as linkers between growth and defense.

### Flavonoids Might Alleviate the Growth Inhibition Caused by Intermittent Wounding in *Arabidopsis*

To decipher the role of flavonoids in plant defense, we tested a flavonoid synthesis mutant, *chs*, for resistance to cotton bollworm. Although the high concentration of flavonoids (kaempferol-3,7-dirhamnoside) in the artificial diet was toxic to *Pieris brassicae* (Onkokesung et al., 2014), *chs* displayed similar resistance to *H. armigera*, *P. xylostella*, and *Spodoptera frugiperda*, as the WT (Supplementary Figure 8A). In addition, the inhibition of root growth and the induction of gene expression by the JA treatment were similar in *chs* and WT (Supplementary Figures 8B–D), indicating that the JA response was not affected in *chs*.

A tradeoff between plant defense and growth has been reported (Major et al., 2017; Wasternack, 2017). Active defense responses usually inhibit plant growth. Some plants exhibit a strong compensatory capacity for growth by increasing the photosynthetic rate and compensatory regrowth, and improving the utilization rate of stored nutrients to compensate for the damage caused by stress (Stowe et al., 2000; Turley et al., 2013). The expression of flavonoid synthesis genes was highly correlated with that of growth-related genes (Figure 5C), which inspired the authors to determine whether flavonoids are involved in growth regulation when plants are under biotic stress.

Herbivorous insect actions are intermittent rather than persistent. To mimic insect feeding, we treated WT and *chs* with intermittent wounding. The plant sizes of the WT and *chs* were similar under normal conditions. After 22 days of intermittent wounding treatment, both types of plants were smaller than the untreated control; and, notably, the *chs* plants were much smaller than those of WT (Figure 5E and Supplementary Figure 9). Consistently, the plant biomass was reduced by intermittent wounding, and the reduction was more significant in *chs* (Figure 5F). These results showed that intermittent wounding caused more significant growth inhibition in *chs* than in WT. A plausible explanation is that flavonoids alleviated the growth inhibition caused by wounding in *Arabidopsis*.

## Discussion

Jasmonate signaling plays an essential role in coordinating plant defense and development. MYC2/MYC3/MYC4 are the main TFs and have functional redundancy in JA signaling, such as plant defense and floral transition (Fernandez-Calvo et al., 2011; Wang et al., 2017; Van Moerkercke et al., 2019). However, the GO enrichment analysis of genes affected only by MYC2, MYC3, or MYC4 revealed the differential functions of individual MYC in many biological processes (Figure 2E). MYC2 might function predominantly in plant growth and development, and flavonoid synthesis is primarily regulated by MYC3, while MYC4 is involved in trichome formation.

We found 94 MGAIs in the untreated plants, suggesting their possible involvement in plant constitutive defense. In addition, the 545 MGAIs in the wounded plants indicated their potential role in wounding-induced defense. Interestingly, only 13 MGAIs in untreated and wounded plants overlapped (Figure 3A and Supplementary Table 7), suggesting that constitutive and wounding-induced defense against cotton bollworm followed different mechanisms in *Arabidopsis*. Among the three single mutants, the expression of MGAIs in *myc3* was the lowest, and the cotton bollworm gained the highest weight when raised on *myc3*, indicating the dominant contribution of MYC3 to plant defense against cotton bollworm.

Phenylpropanoids are abundant phenolic secondary plant metabolites derived from the aromatic amino acid phenylalanine in plants. Inducible phenylpropanoids and their derivatives, such as flavonoids, are tightly coupled with many plant biotic stresses, namely, pathogen infections and insect herbivory (Odonbayar et al., 2016; Ranjan et al., 2019; Vanholme et al., 2019; Seybold et al., 2020). Accumulated flavonoids are believed to affect auxin transport and affect plant growth (Besseau et al., 2007; Dong et al., 2020). Furthermore, higher concentrations of flavonoids have been reported to inhibit the growth of some insects, such as whitefly (Onkokesung et al., 2014; Xia et al., 2021). Flavonoid accumulation could be easily induced by various abiotic or biotic stresses, such as ultraviolet B or temperature (Petrussa et al., 2013). This indicates that flavonoids might have important roles in plants for adaptation to stresses. Although in this study the lack of flavonoids had little effect on *Arabidopsis* resistance to insects, flavonoids might be beneficial in relieving the growth inhibition caused by wounding (Supplementary Figure 8A and Figure 5F). Coupled with the evidence that flavonoid synthesis genes are highly co-expressed with auxin response genes (Figures 5C,D), flavonoids may be implicated in coordinating auxin signaling to help plants recover from wounding or insect attack.

## Data Availability Statement

The original contributions presented in the study are publicly available. This data can be found here: All Illumina sequencing data have been deposited in National Center for Biotechnology Information (NCBI) database: Short Read Archives (SRA) database (BioProject ID PRJNA720476, Run ID 17877327-17877374).

## Author Contributions

D-DW, PL, and Y-BM designed the study, analyzed the data, and wrote the manuscript with the input of all the authors. D-DW performed most of the experiments with the assistance of Q-YC, X-YC, Z-WY, and M-YW. PL performed the sequence analysis. All authors contributed to the article and approved the submitted version.

## Conflict of Interest

The authors declare that the research was conducted in the absence of any commercial or financial relationships that could be construed as a potential conflict of interest.

## Footnotes

1 https://www.arabidopsis.org/

## References

[B1] Agati G. Azzarello E. Pollastri S. Tattini M. (2012). Flavonoids as antioxidants in plants: location and functional significance. *Plant Sci.* 196 67–76. 10.1016/j.plantsci.2012.07.014 23017900

[B2] Anshul N. Bhakuni R. S. Gaur R. Singh D. (2013). Isomeric flavonoids of artemisia annua (Asterales: Asteraceae) as insect growth inhibitors against *Helicoverpa armigera* (Lepidoptera: Noctuidae). *Florida Entomol.* 96 897–903. 10.1653/024.096.0325

[B3] Besseau S. Hoffmann L. Geoffroy P. Lapierre C. Pollet B. Legrand M. (2007). Flavonoid accumulation in *Arabidopsis* repressed in lignin synthesis affects auxin transport and plant growth. *Plant Cell* 19 148–162. 10.1105/tpc.106.044495 17237352 PMC1820963

[B4] Bones A. M. Rossiter J. T. (2006). The enzymic and chemically induced decomposition of glucosinolates. *Phytochemistry* 67 1053–1067. 10.1016/j.phytochem.2006.02.024 16624350

[B5] Brown D. E. Rashotte A. M. Murphy A. S. Normanly J. Tague B. W. Peer W. A. (2001). Flavonoids act as negative regulators of auxin transport in vivo in *Arabidopsis*. *Plant Physiol.* 126 524–535. 10.1104/pp.126.2.524 11402184 PMC111146

[B6] Browse J. (2009). Jasmonate passes muster: a receptor and targets for the defense hormone. *Annu. Rev. Plant Biol.* 60 183–205. 10.1146/annurev.arplant.043008.092007 19025383

[B7] Carretero-Paulet L. Galstyan A. Roig-Villanova I. Martinez-Garcia J. F. Bilbao-Castro J. R. Robertson D. L. (2010). Genome-wide classification and evolutionary analysis of the bHLH family of transcription factors in *Arabidopsis*, poplar, rice, moss, and algae. *Plant Physiol.* 153 1398–1412. 10.1104/pp.110.153593 20472752 PMC2899937

[B8] Chalker-Scott L. (1999). Environmental significance of anthocyanins in plant stress responses. *Photochem. Photobiol.* 70 1–9. 10.1111/j.1751-1097.1999.tb01944.x

[B9] Chen X. Wang D. D. Fang X. Chen X. Y. Mao Y. B. (2019). Plant specialized metabolism regulated by jasmonate signaling. *Plant Cell Physiol.* 60 2638–2647. 10.1093/pcp/pcz161 31418777

[B10] Clayton W. A. Albert N. W. Thrimawithana A. H. McGhie T. K. Deroles S. C. Schwinn K. E. (2018). UVR8-mediated induction of flavonoid biosynthesis for UVB tolerance is conserved between the liverwort Marchantia polymorpha and flowering plants. *Plant J.* 96 503–517. 10.1111/tpj.14044 30044520

[B11] De Geyter N. Gholami A. Goormachtig S. Goossens A. (2012). Transcriptional machineries in jasmonate-elicited plant secondary metabolism. *Trends Plant Sci.* 17 349–359. 10.1016/j.tplants.2012.03.001 22459758

[B12] Dong N. Q. Lin H. X. (2021). Contribution of phenylpropanoid metabolism to plant development and plant-environment interactions. *J. Integr. Plant Biol.* 63 180–209. 10.1111/jipb.13054 33325112

[B13] Dong N. Q. Sun Y. W. Guo T. Shi C. L. Zhang Y. M. Kan Y. (2020). UDP-glucosyltransferase regulates grain size and abiotic stress tolerance associated with metabolic flux redirection in rice. *Nat. Commun.* 11:2629. 32457405 10.1038/s41467-020-16403-5 PMC7250897

[B14] Erb M. Glauser G. (2010). Family business: multiple members of major phytohormone classes orchestrate plant stress responses. *Chemistry* 16 10280–10289. 10.1002/chem.201001219 20648494

[B15] Erb M. Meldau S. Howe G. A. (2012). Role of phytohormones in insect-specific plant reactions. *Trends Plant Sci.* 17 250–259. 10.1016/j.tplants.2012.01.003 22305233 PMC3346861

[B16] Fernandez-Calvo P. Chini A. Fernandez-Barbero G. Chico J. M. Gimenez-Ibanez S. Geerinck J. (2011). The *Arabidopsis* bHLH transcription factors MYC3 and MYC4 are targets of JAZ repressors and act additively with MYC2 in the activation of jasmonate responses. *Plant Cell* 23 701–715. 10.1105/tpc.110.080788 21335373 PMC3077776

[B17] Glauser G. Schweizer F. Turlings T. C. J. Reymond P. (2012). Rapid profiling of intact glucosinolates in *Arabidopsis* leaves by UHPLC-QTOFMS using a charged surface hybrid column. *Phytochem. Anal.* 23 520–528. 10.1002/pca.2350 22323091

[B18] Goossens J. Mertens J. Goossens A. (2017). Role and functioning of bHLH transcription factors in jasmonate signalling. *J. Exp. Bot.* 68 1333–1347. 27927998 10.1093/jxb/erw440

[B19] Halkier B. A. Gershenzon J. (2006). Biology and biochemistry of glucosinolates. *Annu. Rev. Plant Biol.* 57 303–333. 10.1146/annurev.arplant.57.032905.105228 16669764

[B20] Howe G. A. Jander G. (2008). Plant immunity to insect herbivores. *Annu. Rev. Plant Biol.* 59 41–66. 10.1146/annurev.arplant.59.032607.092825 18031220

[B21] Howe G. A. Major I. T. Koo A. J. (2018). Modularity in jasmonate signaling for multistress resilience. *Annu. Rev. Plant Biol.* 69 387–415. 10.1146/annurev-arplant-042817-040047 29539269

[B22] Jiang W. B. Yin Q. G. Wu R. R. Zheng G. S. Liu J. Y. Dixon R. A. (2015). Role of a chalcone isomerase-like protein in flavonoid biosynthesis in *Arabidopsis thaliana*. *J. Exp. Bot.* 66 7165–7179. 10.1093/jxb/erv413 26347569 PMC4765788

[B23] Kazan K. Manners J. M. (2013). MYC2: the master in action. *Mol. Plant* 6 686–703. 10.1093/mp/sss128 23142764

[B24] Langfelder P. Horvath S. (2008). WGCNA: an R package for weighted correlation network analysis. *BMC Bioinform.* 9:559. 10.1186/1471-2105-9-559 19114008 PMC2631488

[B25] Liang T. Shi C. Peng Y. Tan H. J. Xin P. Y. Yang Y. (2020). Brassinosteroid-activated BRI1-EMS-SUPPRESSOR 1 inhibits flavonoid biosynthesis and coordinates growth and UV-B stress responses in plants. *Plant Cell* 32 3224–3239. 10.1105/tpc.20.00048 32796123 PMC7534464

[B26] Love M. I. Huber W. Anders S. (2014). Moderated estimation of fold change and dispersion for RNA-seq data with DESeq2. *Genome Biol.* 15:550. 25516281 10.1186/s13059-014-0550-8 PMC4302049

[B27] Major I. T. Yoshida Y. Campos M. L. Kapali G. Xin X. F. Sugimoto K. (2017). Regulation of growth-defense balance by the JASMONATE ZIM-DOMAIN (JAZ)-MYC transcriptional module. *New Phytol.* 215 1533–1547. 10.1111/nph.14638 28649719 PMC5542871

[B28] Manzaneda A. J. Prasad K. V. S. K. Mitchell-Olds T. (2010). Variation and fitness costs for tolerance to different types of herbivore damage in Boechera stricta genotypes with contrasting glucosinolate structures. *New Phytol.* 188 464–477. 10.1111/j.1469-8137.2010.03385.x 20663059 PMC2950872

[B29] Mewis I. Tokuhisa J. G. Schultz J. C. Appel H. M. Ulrichs C. Gershenzon J. (2006). Gene expression and glucosinolate accumulation in *Arabidopsis thaliana* in response to generalist and specialist herbivores of different feeding guilds and the role of defense signaling pathways. *Phytochemistry* 67 2450–2462. 10.1016/j.phytochem.2006.09.004 17049571

[B30] Odonbayar B. Murata T. Batkhuu J. Yasunaga K. Goto R. Sasaki K. (2016). Antioxidant flavonols and phenolic compounds from atraphaxis frutescens and their inhibitory activities against insect phenoloxidase and mushroom tyrosinase. *J. Nat. Prod.* 79 3065–3071. 10.1021/acs.jnatprod.6b00720 28006914

[B31] Okada K. Abe H. Arimura G. (2015). Jasmonates induce both defense responses and communication in *Monocotyledonous* and *Dicotyledonous* plants. *Plant and Cell Physiology* 56 16–27. 10.1093/pcp/pcu158 25378688

[B32] Onkokesung N. Reichelt M. van Doorn A. Schuurink R. C. van Loon J. J. A. Dicke M. (2014). Modulation of flavonoid metabolites in *Arabidopsis thaliana* through overexpression of the MYB75 transcription factor: role of kaempferol-3,7-dirhamnoside in resistance to the specialist insect herbivore *Pieris brassicae*. *J. Exp. Bot.* 65 2203–2217. 10.1093/jxb/eru096 24619996 PMC3991749

[B33] Petrussa E. Braidot E. Zancani M. Peresson C. Bertolini A. Patui S. (2013). Plant flavonoids-biosynthesis, transport and involvement in stress responses. *Int. J. Mol. Sci.* 14 14950–14973. 10.3390/ijms140714950 23867610 PMC3742282

[B34] Ranjan A. Westrick N. M. Jain S. Piotrowski J. S. Ranjan M. Kessens R. (2019). Resistance against Sclerotinia sclerotiorum in soybean involves a reprogramming of the phenylpropanoid pathway and up-regulation of antifungal activity targeting ergosterol biosynthesis. *Plant Biotechnol. J.* 17 1567–1581. 10.1111/pbi.13082 30672092 PMC6662107

[B35] Schlaeppi K. Bodenhausen N. Buchala A. Mauch F. Reymond P. (2008). The glutathione-deficient mutant pad2-1 accumulates lower amounts of glucosinolates and is more susceptible to the insect herbivore Spodoptera littoralis. *Plant J.* 55 774–786. 10.1111/j.1365-313x.2008.03545.x 18466300

[B36] Schweizer F. Bodenhausen N. Lassueur S. Masclaux F. G. Reymond P. (2013a). Differential contribution of transcription factors to *Arabidopsis thaliana* defense against *Spodoptera littoralis*. *Front. Plant Sci.* 4:13. 10.3389/fpls.2013.00013 23382734 PMC3563046

[B37] Schweizer F. Fernandez-Calvo P. Zander M. Diez-Diaz M. Fonseca S. Glauser G. (2013b). *Arabidopsis* basic helix-loop-helix transcription factors MYC2, MYC3, and MYC4 regulate glucosinolate biosynthesis, insect performance, and feeding behavior. *Plant Cell* 25 3117–3132. 10.1105/tpc.113.115139 23943862 PMC3784603

[B38] Senecoff J. F. McKinney E. C. Meagher R. B. (1996). De novo purine synthesis in *Arabidopsis thaliana*. 2. The PUR7 gene encoding 5’-phosphoribosyl-4-(N-succinocarboxamide)-5-aminoimidazole synthetase is expressed in rapidly dividing tissues. *Plant Physiol.* 112 905–917. 10.1104/pp.112.3.905 8938402 PMC158018

[B39] Seybold H. Demetrowitsch T. J. Hassani M. A. Szymczak S. Reim E. Haueisen J. (2020). A fungal pathogen induces systemic susceptibility and systemic shifts in wheat metabolome and microbiome composition. *Nat. Commun.* 11:1910. 32313046 10.1038/s41467-020-15633-x PMC7171108

[B40] Sharma A. Badola P. K. Bhatia C. Sharma D. Trivedi P. K. (2020). Primary transcript of miR858 encodes regulatory peptide and controls flavonoid biosynthesis and development in *Arabidopsis*. *Nat. Plants* 6 1262–1274. 10.1038/s41477-020-00769-x 32958895

[B41] Simmonds M. S. J. Stevenson P. C. (2001). Effects of isoflavonoids from Cicer on larvae of Heliocoverpa armigera. *J. Chem. Ecol.* 27 965–977. 11471948 10.1023/a:1010339104206

[B42] Song S. S. Huang H. Gao H. Wang J. J. Wu D. W. Liu X. L. (2014). Interaction between MYC2 and ETHYLENE INSENSITIVE3 modulates antagonism between jasmonate and ethylene signaling in *Arabidopsis*. *Plant Cell* 26 263–279. 10.1105/tpc.113.120394 24399301 PMC3963574

[B43] Stowe K. A. Marquis R. J. Hochwender C. G. Simms E. L. (2000). The evolutionary ecology of tolerance to consumer damage. *Annu. Rev. Ecol. Syst.* 31 565–595. 10.1146/annurev.ecolsys.31.1.565

[B44] Stracke R. Favory J. J. Gruber H. Bartelniewoehner L. Bartels S. Binkert M. (2010). The *Arabidopsis* bZIP transcription factor HY5 regulates expression of the PFG1/MYB12 gene in response to light and ultraviolet-B radiation. *Plant Cell Environ.* 33 88–103. 10.1111/j.1365-313x.2011.04670.x 19895401

[B45] Tan H. Man C. Xie Y. Yan J. Chu J. Huang J. (2019). A crucial role of GA-regulated flavonol biosynthesis in root growth of *Arabidopsis*. *Mol. Plant* 12 521–537. 10.1016/j.molp.2018.12.021 30630075

[B46] Tenenbaum D. Maintainer B. P. (2020). *KEGGREST: Client-side REST access to the Kyoto Encyclopedia of Genes and Genomes (KEGG). R package version 1.33.0.*

[B47] Turley N. E. Godfrey R. M. Johnson M. T. J. (2013). Evolution of mixed strategies of plant defense against herbivores. *New Phytol.* 197 359–361. 10.1111/nph.12103 23253329

[B48] Van Moerkercke A. Duncan O. Zander M. Simura J. Broda M. Vanden Bossche R. (2019). A MYC2/MYC3/MYC4-dependent transcription factor network regulates water spray-responsive gene expression and jasmonate levels. *Proc. Natl. Acad. Sci. U.S.A.* 116 23345–23356. 10.1073/pnas.1911758116 31662474 PMC6859355

[B49] Vanholme B. El Houari I. Boerjan W. (2019). Bioactivity: phenylpropanoids’ best kept secret. *Curr. Opin. Biotechnol.* 56 156–162. 10.1016/j.copbio.2018.11.012 30530240

[B50] Wang H. Li S. Y. Li Y. A. Xu Y. R. Wang Y. H. Zhang R. X. (2019). MED25 connects enhancer-promoter looping and MYC2-dependent activation of jasmonate signalling. *Nat. Plants* 5 616–625. 10.1038/s41477-019-0441-9 31182849

[B51] Wang H. P. Li Y. Pan J. J. Lou D. J. Hu Y. R. Yu D. Q. (2017). The bHLH transcription factors MYC2, MYC3, and MYC4 are required for jasmonate-mediated inhibition of flowering in *Arabidopsis*. *Mol. Plant* 10 1461–1464. 10.1016/j.molp.2017.08.007 28827172

[B52] Wasternack C. (2017). A plant’s balance of growth and defense - revisited. *New Phytol.* 215 1291–1294. 10.1111/nph.14720 28771818

[B53] Wasternack C. Hause B. (2013). Jasmonates: biosynthesis, perception, signal transduction and action in plant stress response, growth and development. An update to the 2007 review in Annals of Botany. *Ann. Bot.* 111 1021–1058. 10.1093/aob/mct067 23558912 PMC3662512

[B54] Wasternack C. Strnad M. (2019). Jasmonates are signals in the biosynthesis of secondary metabolites - Pathways, transcription factors and applied aspects - A brief review. *N. Biotechnol.* 48 1–11. 10.1016/j.nbt.2017.09.007 29017819

[B55] Wen W. W. Alseekh S. Fernie A. R. (2020). Conservation and diversification of flavonoid metabolism in the plant kingdom. *Curr. Opin. Plant Biol.* 55 100–108. 10.1016/j.pbi.2020.04.004 32422532

[B56] Wiszniewski A. A. Zhou W. Smith S. M. Bussell J. D. (2009). Identification of two *Arabidopsis* genes encoding a peroxisomal oxidoreductase-like protein and an acyl-CoA synthetase-like protein that are required for responses to pro-auxins. *Plant Mol. Biol.* 69 503–515. 10.1007/s11103-008-9431-4 19043666

[B57] Wittstock U. Gershenzon J. (2002). Constitutive plant toxins and their role in defense against herbivores and pathogens. *Curr. Opin. Plant Biol.* 5 300–307. 10.1016/s1369-5266(02)00264-9 12179963

[B58] Xia J. Guo Z. Yang Z. Han H. Wang S. Xu H. (2021). Whitefly hijacks a plant detoxification gene that neutralizes plant toxins. *Cell* 184 1693.e17–1705.e17. 33770502 10.1016/j.cell.2021.02.014

[B59] Yang J. Duan G. H. Li C. Q. Liu L. Han G. Y. Zhang Y. L. (2019). The crosstalks between jasmonic acid and other plant hormone signaling highlight the involvement of jasmonic acid as a core component in plant response to biotic and abiotic stresses. *Front. Plant Sci.* 10:1349. 10.3389/fpls.2019.01349 31681397 PMC6813250

[B60] Yu G. C. Wang L. G. Han Y. Y. He Q. Y. (2012). clusterProfiler: an R package for comparing biological themes among gene clusters. *Omics J. Integr. Biol.* 16 284–287. 10.1089/omi.2011.0118 22455463 PMC3339379

[B61] Zander M. Lewsey M. G. Clark N. M. Yin L. L. Bartlett A. Guzman J. P. S. (2020). Integrated multi-omics framework of the plant response to jasmonic acid. *Nat. Plants* 6 290–297. 10.1038/s41477-020-0605-7 32170290 PMC7094030

[B62] Zhang X. He Y. Li L. Liu H. Hong G. (2021). Involvement of the R2R3-MYB transcription factors MYB21 and its homologs in regulating the stamen flavonols accumulation in *Arabidopsis*. *J. Exp. Bot.* 72 4319–4332. 10.1093/jxb/erab156 33831169 PMC8163065

[B63] Zhou M. L. Memelink J. (2016). Jasmonate-responsive transcription factors regulating plant secondary metabolism. *Biotechnol. Adv.* 34 441–449. 10.1016/j.biotechadv.2016.02.004 26876016

[B64] Zubo Y. O. Blakley I. C. Franco-Zorrilla J. M. Yamburenko M. V. Solano R. Kieber J. J. (2018). Coordination of chloroplast development through the action of the GNC and GLK transcription factor families. *Plant Physiol.* 178 130–147. 10.1104/pp.18.00414 30002259 PMC6130010

