# Supplementary material for: Differential Contributions of MYCs to Insect Defense Reveals Flavonoids Alleviating Growth Inhibition Caused by Wounding in Arabidopsis
Source: Front Plant Sci. 2021 Jul 13;12:700555. doi: 10.3389/fpls.2021.700555 (PMC8313826; doi:10.3389/fpls.2021.700555)
Supplement: Supplementary file 1 [file Data_Sheet_1.docx]

***Supplementary Material***

**Supplementary Figure S1.** Gene expressions of the selected MYC-affected genes.

**Supplementary Figure S2.** MYC-affected genes and their GO annotation.

**Supplementary Figure S3.** Number of MGAIs in untreated and wounded plants.

**Supplementary Figure S4.** Glucosinolates are positively related to plant resistance against cotton bollworm.

**Supplementary Figure S5.** Venn diagrams of MGAIs and genes which were affected by MYCs.

**Supplementary Figure S6.** Modules cluster analysis of 48 groups’ transcriptome data.

**Supplementary Figure S7.** Co-expression network analysis of genes classified as dark magenta.

**Supplementary Figure S8.** JA response were not influenced in *chs*.

**Supplementary Figure S9.** Images of the plants by intermittent wounding treatments.

**Supplementary Table S1** Primers used in this research.

**Supplementary Table S2** Detailed gene information of MYC-affected genes in untreated and wounded samples as shown in Figure 2B.

**Supplementary Table S3** GO enrichment analysis of MYC-affected genes in untreated and wounded plants, as described in Figure 2C.

**Supplementary Table S4** List of MYC2, MYC3, and MYC4-affected genes in untreated and wounded plants, as described in Figure 2D and Supplementary Figure S2A.

**Supplementary Table S5** GO enrichment analysis of MYC2-, MYC3-, and MYC4-affected genes, as described in Figure 2E and Supplementary Figure S2B.

**Supplementary Table S6** Genes highly correlated with insect resistance in untreated and wounded plants, as described in Supplementary Figure S3.

**Supplementary Table S7** List of MGAI genes in untreated and wounded plants in Figure 3A.

**Supplementary Table S8** KEGG enrichment analysis of MGAIs in untreated and wounded plants, as described in Figure 3B.

**Supplementary Table S9** GLS-related genes Z-scored TPM in mycs-related mutants in untreated and wounded plants as described in Supplementary Figure S4A.

**Supplementary Table S10** MGAIs affected by MYCs as described in Supplementary Figure S5.

**Supplementary Table S11** MGAIs Z-scored TPM in mycs-related mutants in untreated and wounded plants, as described in Figure 3 D and E.

**Supplementary Table S12** Gene distributions in the enriched modules of MYC-affected genes found in untreated and wounded plants (Figure 4 B and C).

**Supplementary Table S13** GO enrichment analysis of MYC-affected genes in enriched modules, as described in Figure 4 D and E.

**Supplementary Table S14** Detailed information of the Z-scored TPMs of Flavonoid synthesis genes in mycs-related mutants in Figure 5B.

**Supplementary Table S 2 to 14:** See separate Excel file.

Supplementary Figure


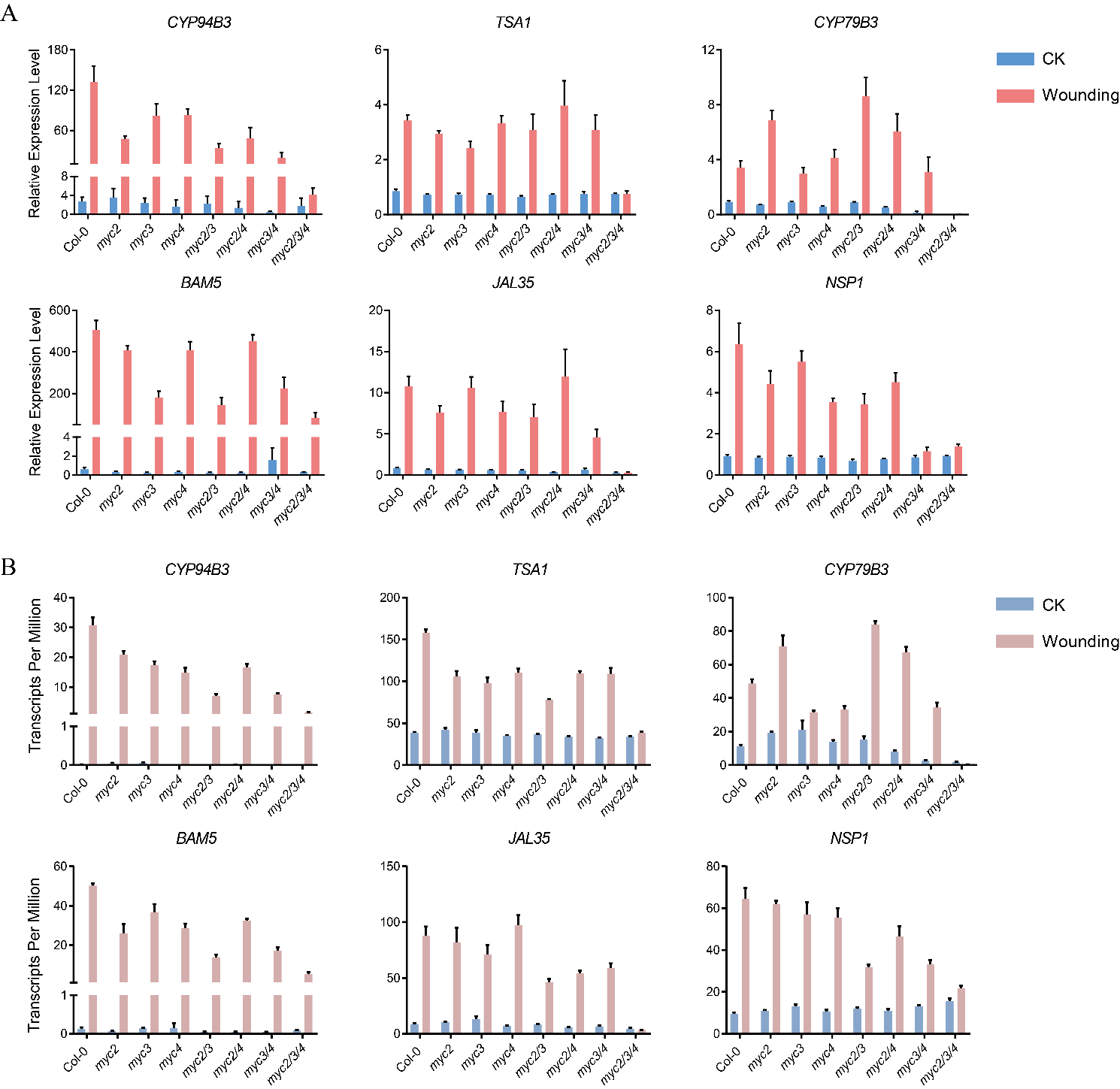


**Supplementary Figure S1. Gene expressions of the selected MYC-affected genes.** Data are mean ± SEM (n=3). (A) Gene expressions were detected by RT-qPCR. The wild type (Col-0) and the different myc-related mutants were wounded and harvested 4 h post wounding. The corresponding untreated plants were used as controls (CK). *S18* was used as the internal standard. The expression in untreated Col-0 were set to 1. (B) The TPM (Transcripts Per Million) of the selected genes in RNA-seq data.


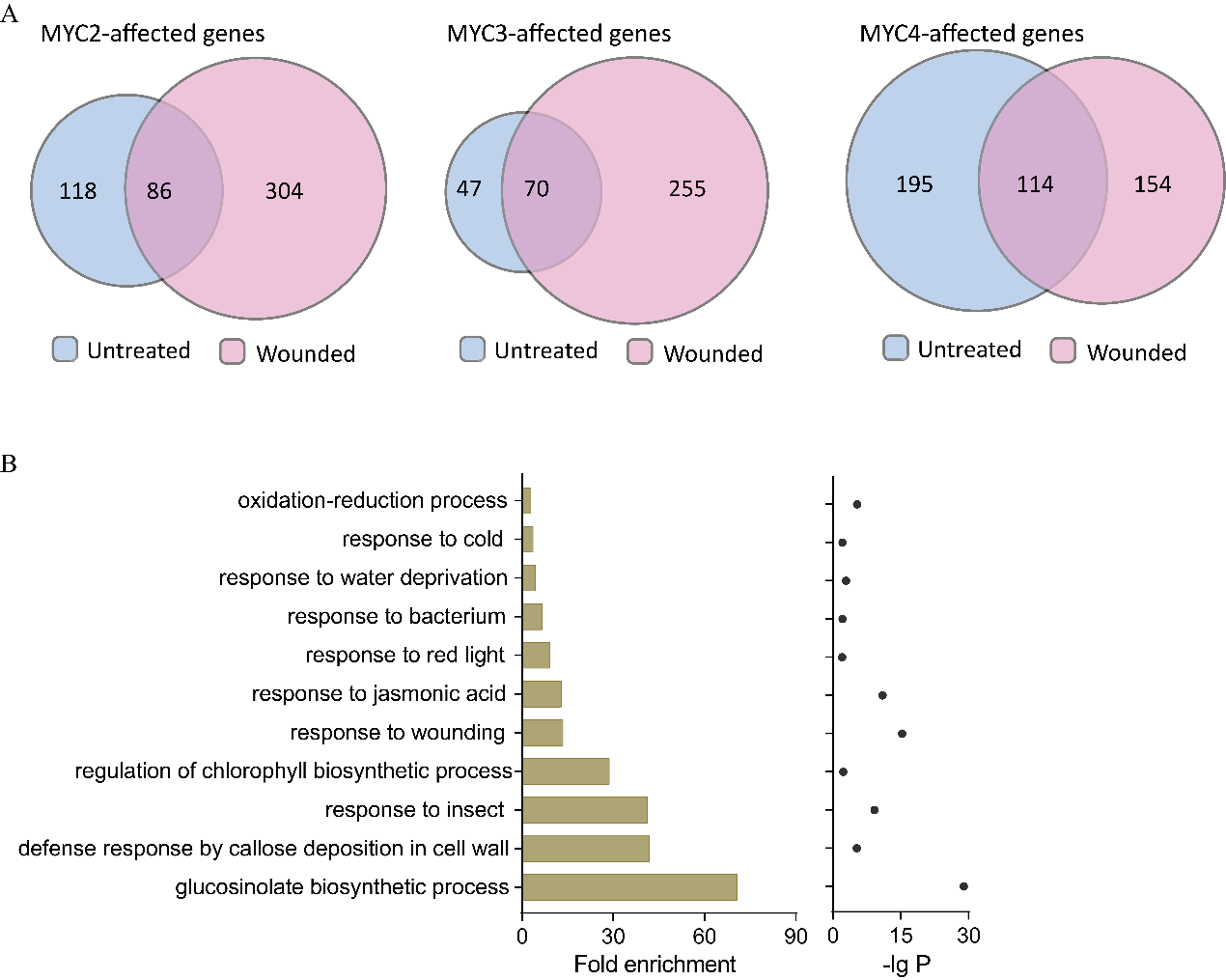


**Supplementary Figure S2. MYCs-affected genes and its GO annotation.** The detailed gene information was presented in Supplementary Table S4. (A) Differentially expressed genes (DEGs) which were affected by MYC2, MYC3 and MYC4. Blue and red stand for genes affected by indicated MYC in untreated and wounded plants respectively. (B) GO enrichment analysis of 172 genes obtained from Figure 2D of which expressions were significantly affected by all the three MYCs.


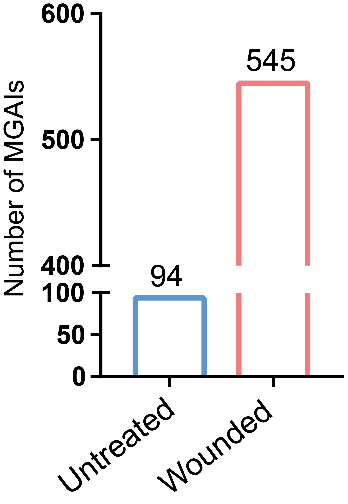


**Supplementary Figure S3. Number of MGAIs in untreated and wounded plants.**


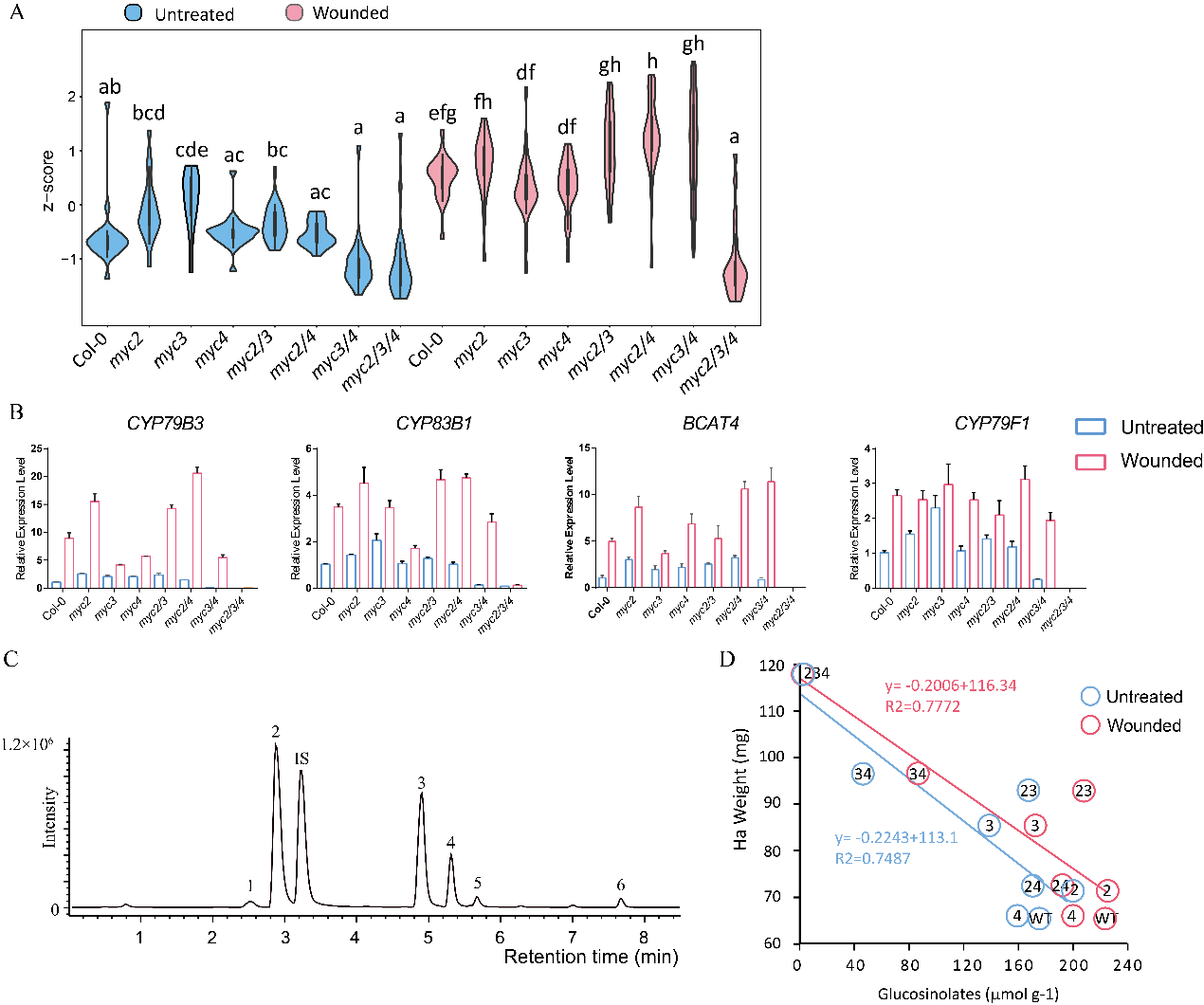


**Supplementary Figure S4. Glucosinolates are positively related to plant resistance against cotton bollworm.**

(A) The expressions of glucosinolate biosynthetic genes in untreated (blue) and wounded (red) plants from the RNA-seq analysis. Genes with GO annotation of glucosinolate biosynthetic process were analyzed. The TPM of each gene was normalized by z-score. The average z-score of each gene was showed in the violin plot black line. The detailed GLSs gene information was presented in Supplementary Table S9. Data were analyzed by one-way ANOVA and Tukey’s HSD test. Different letters indicate significant differences, P-value <0.05. (B) Expressions of indicated genes in glucosinolate biosynthetic process by RT-qPCR analysis. Blue represents untreated plants and red represents plants 4 hours post wounding (wounded). (C) LC-MS chromatogram of glucosinolates in *Arabidopsis* (Col-0) leaves. 1: 4-Hydroxybenzyl-glucosinolate; 2: 4-Methylsulphinylbutyl-glucosinolate, 4MSOB; 3: 4-Methylthiobutyl-glucosinolate, 4MTB; 4: Indol-3-ylmethyl-glucosinolate, I3M; 5: Methoxyindol-3-ylmethyl-glucosinolate, MOI3M; 6: 8-Methylsulphinyloctyl-glucosinolate, 8MSOO; IS: Internal Standard, Sinigrin. (D) Negative relation between the total GLSs content in plant and the growth of *H. armigera* larvae. The contents of 4MTB, 4MSOB, 8MSOO, and I3M were calculated as total GLS. The untreated plants (blue) and the wounded plants (red) which were 36 hours post wounding were harvested for GLS detection.


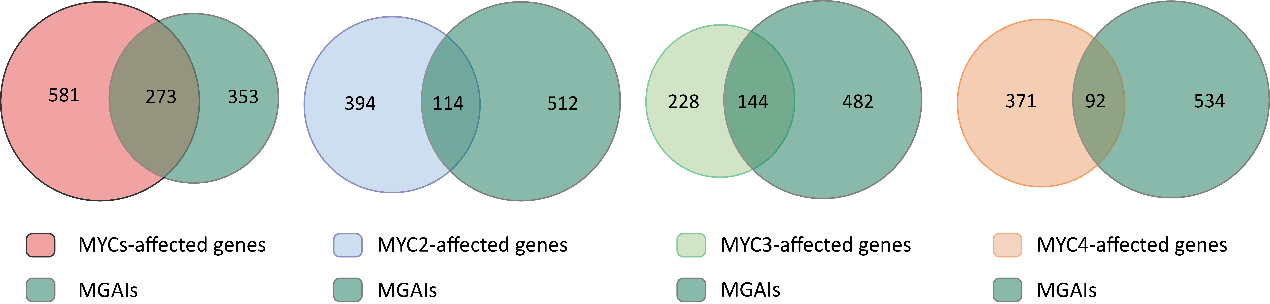


**Supplementary Figure S5. Venn diagrams of MGAIs and genes which were affected by MYCs.** Cyan stands for the total MGAIs either in untreated or wounded plants as described in Figure 3A. Red stand for MYCs-affected genes as described in Figure 2B. Blue, Green, and Orange stand for MYC2, MYC3, and MYC4 affected genes as described in Figure 2D and Supplementary Table S10.


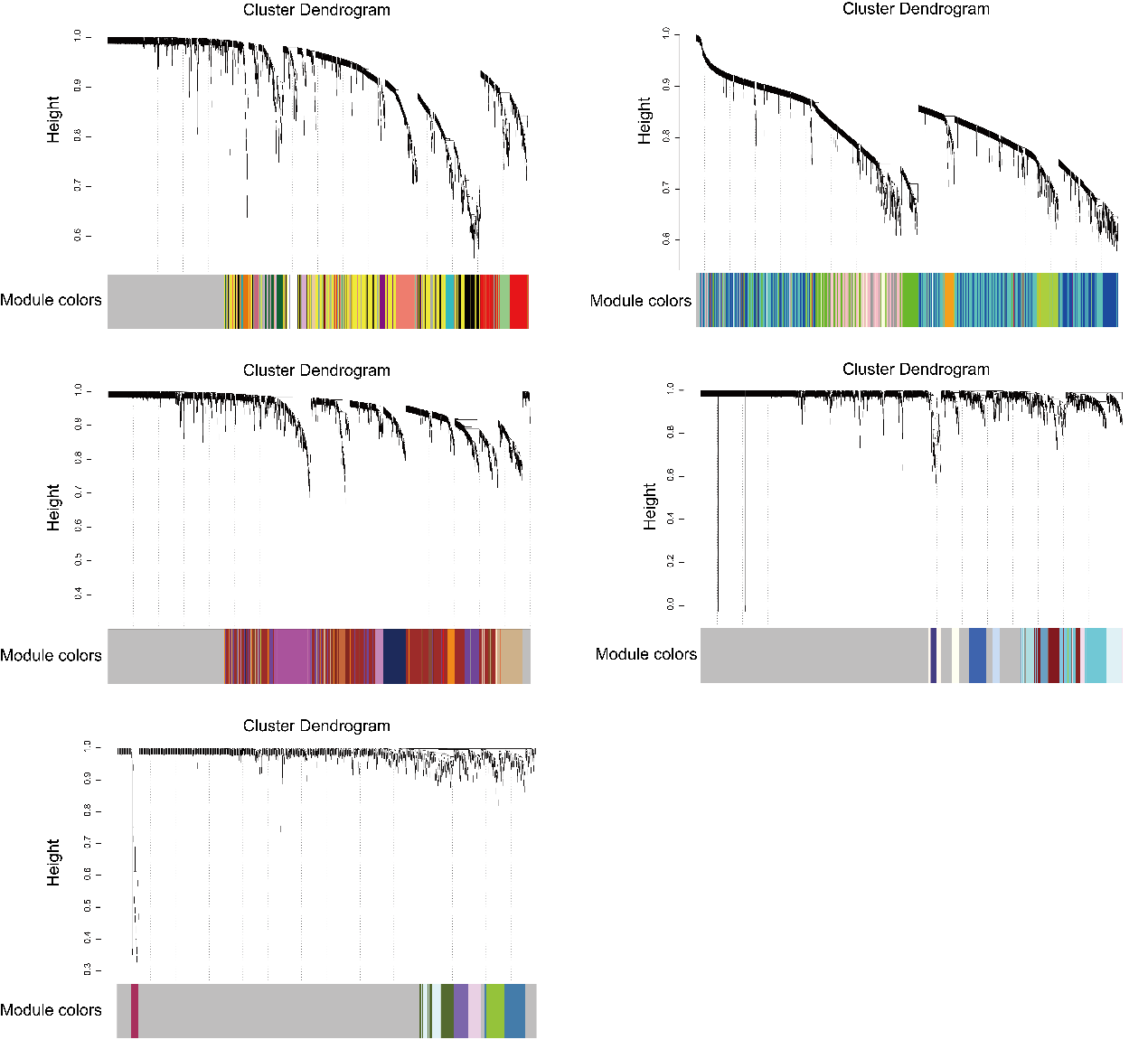


**Supplementary Figure S6. Modules cluster analysis of 48 groups transcriptome data.**


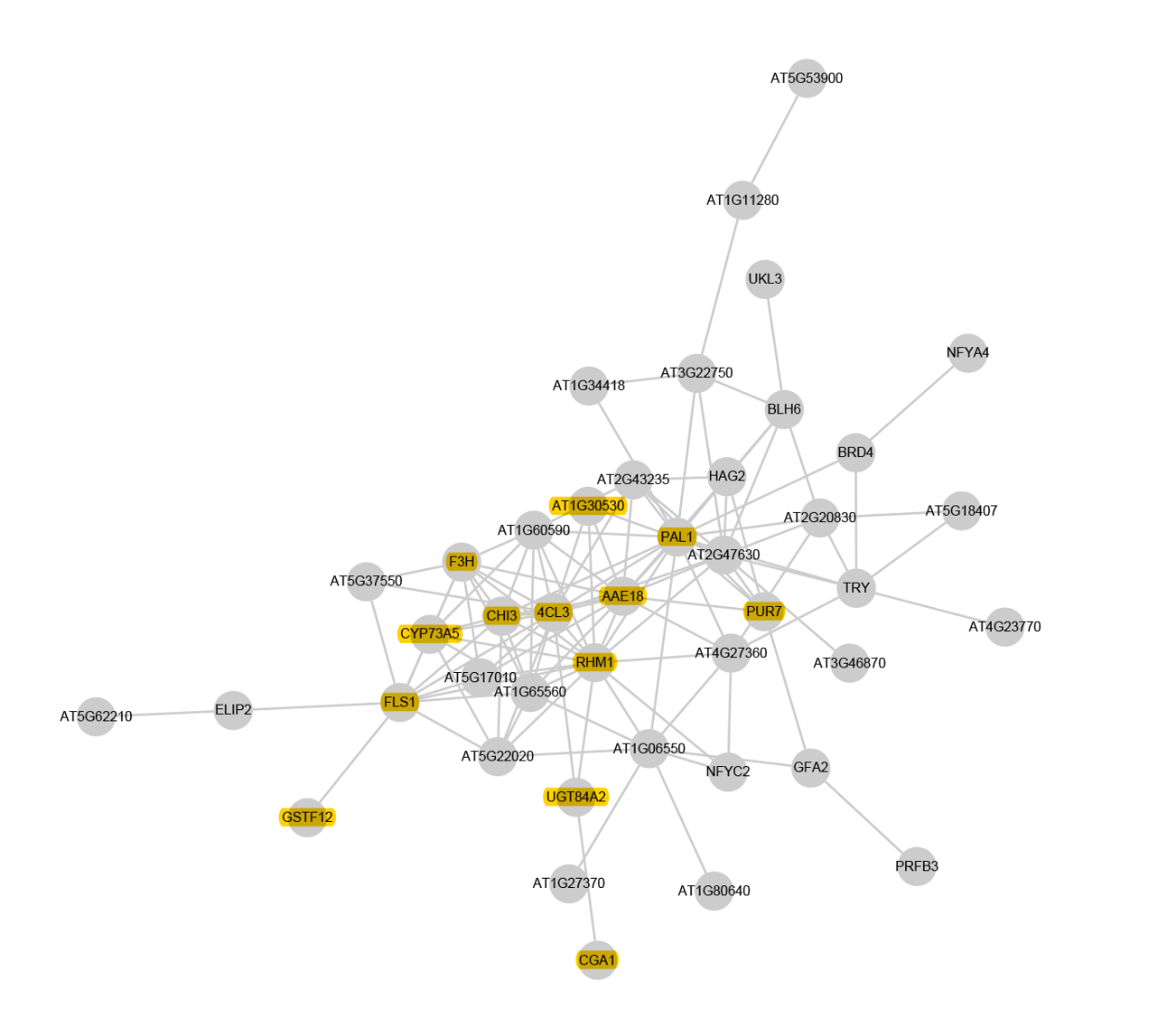


**Supplementary Figure S7. Co-expression network analysis of darkmagenta genes.** Flavonoids synthesis and auxin response genes were highlighted.


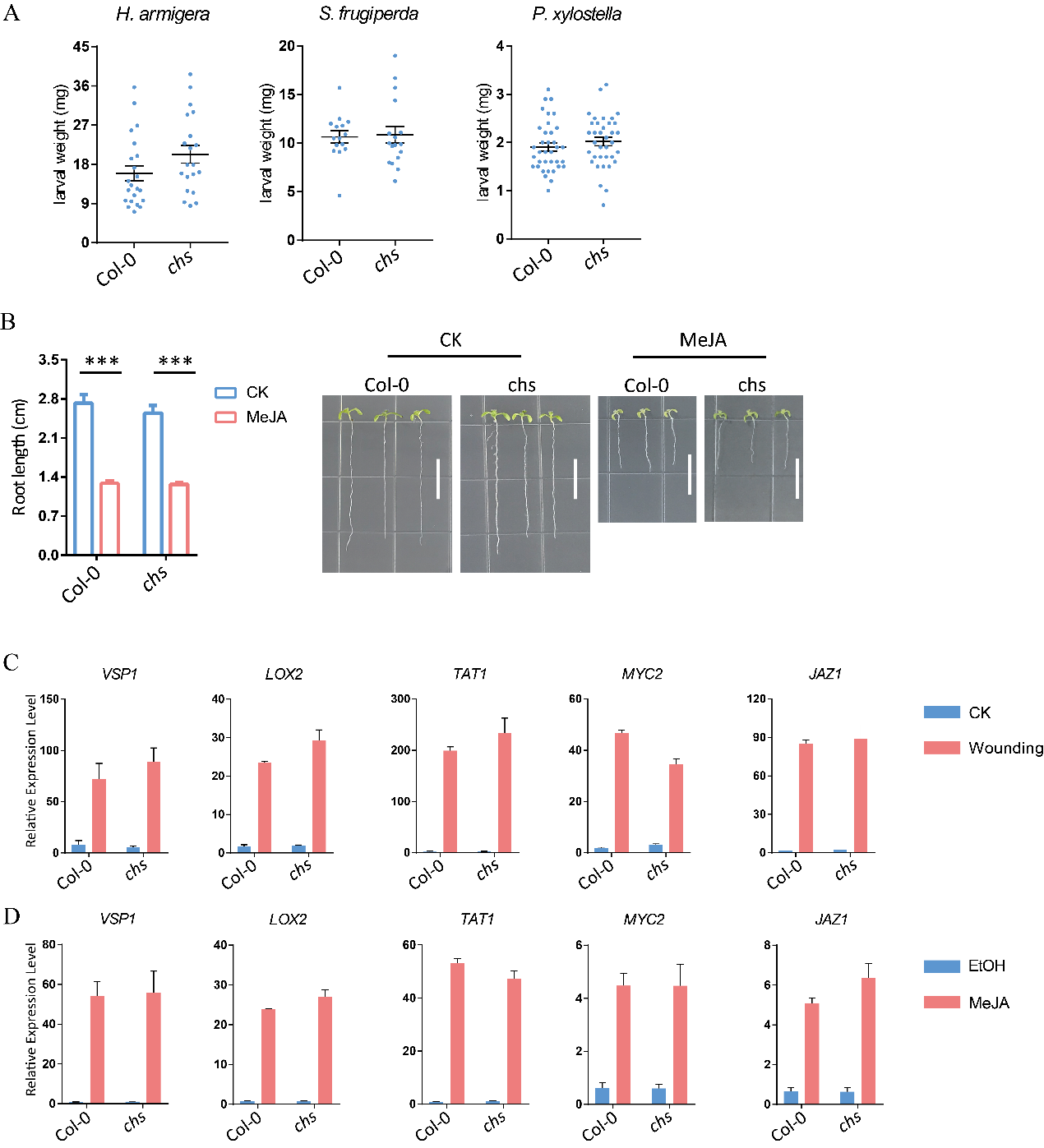


**Supplementary Figure S8. JA response were not influenced in *chs*.** (A) Weight increase of the larvae fed with Col-0 and *chs* respectively. The 2^nd^ instar larvae of *Helicoverpa armigera* and *Spodoptera frugiperda* as well as the *Plutella xylostella* larvae 2 days post hatch were fed with plant leaves for 4 days. (B) Root growth inhibition of Col-0 and *chs* by MeJA treatment. Plant grew in 1/2 MS medium containing 5 μM MeJA or the equal volumes of EtOH for 8 days. Data are means ± SEM (n=15) analyzed by two-way ANOVA and Tukey’s HSD test. *** P-value<0.001. Scale bar, 1 cm. (C-D) Expressions of JA response genes in Col-0 and *chs* with wounding and MeJA treatment by qRT-PCR analysis. *S18* was used as the internal standard. Data are mean ± SEM (n=3). (C) Plants were wounded and harvested two hours post wounding. The expression in untreated plants (CK) were set to 1. (D) Plants were sprayed with 50 μM MeJA and harvested two hours post treatment. The expression in the plants treated with equal volumes of EtOH were set to 1.
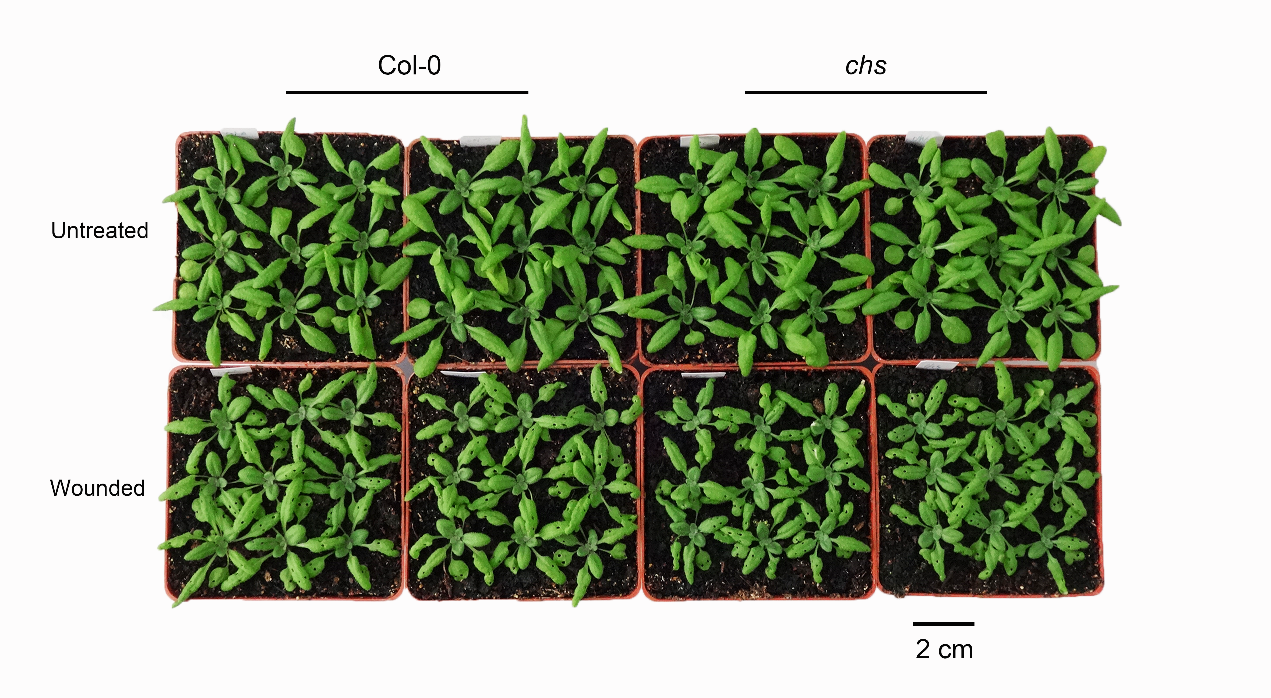


**Supplementary Figure S9. Images of the plants by intermittent wounding treatments.** When plants had grown for 12 days, the first wounding treatment was performed and the wounding treatment was performed every 3 days and lasted for 3 times. Scale bar, 2 cm.

**Supplementary Table S1** Primers used in this research.

| Primer for qPCR. | | |
| --- | --- | --- |
|  | Primer F (5’-3’) | Primer R (5’-3’) |
| AtS18 | CCAGCGATCGTTTATTGCTT | AGTCTTTCCTCTGCGACCAG |
| VSP1 | TCACTACTCTTGCTCTTGGC | CATTGACGGTATCGTTCTTT |
| LOX2 | TTGGTGTGGTAACTACGATTGC | CACCAGCTCCAGCTCTATTCTT |
| TAT1 | CCCTCAAAGACGTCAATGGT | ACACGACACGACAAGTCCAA |
| MYC2 | CAAGGAGGAGTGTTTGGGATGC | GTCGAAAAATTAAGTTCTCGGGAG |
| JAZ1 | GGAGATTTACTGGGAAGAAGCC | GTTTGCGATAGTAGCGATGTTG |
| CYP79B3 | CGATGCTTACGGGATTGGA | CGGCGTTTGATGGGTTGT |
| CYP83B1 | TATTGTTGTGCCGGGAACTG | CCACCTATCTTTGCGTCTGC |
| BCAT4 | GCGAAGTGCAATCATGGAGA | CGAATTGATCGACCGAAGG |
| CYP79F1 | ATCTTGCCAGCTTCCTCCTG | TGCCAAATCTGCGTCTCG |
| CYP94B3 | TTGTAAAGTCTCGTTGGGTTGG | TCTTGCTTTGCTTCTGCTCC |
| TSA1 | GTGGTTCCCGATGTTCCTCT | GCTTGTCTGTCGCCTCTTTG |
| BAM5 | GAAGCCCTAAGTGCTCCTCAA | TCGCTGCGTCTCCACAATA |
| JAL35 | AGATGGTGGAGTTGCTTGGG | CCGACACGGCAGTGACATAC |
| NSP1 | ACCCACTCCTCGTAGTTTCCA | TGCTCCTCCTCTTGCGGTA |
